# Decomposition of Heart Failure Prevalence and Mortality Among Older Adults in the United States: Medicare-Based Partitioning Analysis

**DOI:** 10.2196/51989

**Published:** 2024-11-20

**Authors:** Bin Yu, Julia Kravchenko, Arseniy Yashkin, Igor Akushevich

**Affiliations:** 1 Department of Epidemiology and Health Statistics, School of Public Health, Wuhan University, 115 Donghu Road, Wuchang District, Wuhan, 430071, China, 86 13797095040; 2 Department of Surgery, School of Medicine, Duke University, Durham, NC, United States; 3 Social Science Research Institute, Duke University, Durham, NC, United States

**Keywords:** heart failure, prevalence, mortality, partitioning, time trends, epidemiologic determinants

## Abstract

**Background:**

Heart failure (HF) is a challenging clinical and public health problem characterized by high prevalence and mortality among US older adults, along with a recent decline in HF prevalence and increase in mortality. The changes of prevalence can be decomposed into pre-existing disease prevalence, disease incidence, and respective survival, while the changes of mortality can be decomposed into mortality in the general population independent from HF, pre-existing HF prevalence, incidence, and respective survival. These epidemiological components may contribute differently to the changes in prevalence and mortality.

**Objective:**

We aimed to investigate and compare the relative contributions of epidemiologic determinants in HF prevalence and mortality trends.

**Methods:**

This study was a secondary data analysis of 5% of Medicare claims data for 1992‐2017 in the United States. Medicare is a federal health insurance program for older adults aged 65+ years as well as people with specific disabilities and end-stage renal disease. Age-adjusted prevalence and incidence-based mortality (IBM; all-cause mortality that occurred in patients with HF) were partitioned into their respective epidemiologic determinants using the partitioning analysis approach.

**Results:**

The age-adjusted HF prevalence (1/100 person-years) increased from 11 in 1994 to 14.6 in 2005, followed by a decline to 12.6 in 2017, and the age-adjusted HF IBM (1/100,000) increased from 2220.8 in 1994 to 2563.7 in 2000, then declined to 2075.9 in 2016, followed by an increase to 2094.7 in 2017. The HF incidence (1/1000 person-years) declined from 29.4 in 1992 to 19.9 in 2017. The 1-, 3-, and 5-year survival trend showed declines in recent years. Partitioning of HF prevalence showed three phases: (1) decelerated increasing prevalence (1994‐2006), (2) accelerated declining prevalence (2007‐2014), and (3) decelerated declining prevalence (2015‐2017). During the whole period, the decreasing HF incidence contributed to the declines in prevalence, overpowering prevalence increases contributed from survival. Likewise, partitioning of HF IBM showed three phases: (1) decelerated increasing mortality (1994‐2001), (2) accelerated declining mortality (2002‐2012), and (3) decelerated declining mortality (2013‐2017). The decreasing HF incidence in 1994‐2017 and increasing survival in 2002‐2006 contributed to the declines in mortality, while the decreasing survival in 2007‐2017 contributed to the mortality increase.

**Conclusions:**

Decade-long declines in HF prevalence and mortality mainly reflected decreasing incidence, while the most recent increase of mortality was predominantly due to the declining survival. If current trends persist, HF prevalence and mortality are forecasted to grow substantially in the next decade. Prevention strategies should continue the prevention of HF risk factors as well as improvement of treatment and management of HF after diagnosis.

## Introduction

Heart failure (HF) is a chronic progressive condition that happens when the heart cannot pump enough blood to the organs in the body as a result of structural or functional impairment in ventricular filling or blood ejection [1,2], which remains a challenging clinical and public health problem in the United States [3] and worldwide [2]. An estimated 13.4% of the US total deaths were caused by HF, and nearly 6.2 million adults ever had an incidence of HF [4]. Meanwhile, 6.9% of males and 4.8% of females aged 60‐79 years and 12.8% of males and 12% of females aged 80+ years ever had an incidence of HF [5]. Driven by the population aging [6], HF prevalence is forecasted to increase in the next decade and will surpass the increases of other cardiovascular diseases [7]. Further, the decade-long decline in HF mortality in the United States was reversed in 2012 and continued to grow thereafter [8,9]. According to the most recent data released by the US Centers for Disease Control and Prevention (CDC), the HF mortality in 2022 was 21 per 100,000 (that was 17.1 per 100,000 in 2012)—24.2 per 100,000 for males and 18.5 per 100,000 for females—and was highest in Black (25.8 per 100,000) and lowest in Asian (8.2 per 100,000) individuals [10]. Thus, it is of great significance to quantitatively evaluate the determinants driving these trends.

Several epidemiological components contribute to the observed disease prevalence, including pre-existing disease prevalence, disease incidence, and respective survival [11,12]. Reducing the pre-existing prevalence and incidence can decrease the prevalence, while increasing the survival can increase the prevalence. Prevalence alone cannot differentiate the underlying determinants of the change. Likewise, incidence-based mortality (IBM), all-cause mortality for patients with HF, consists of several components, including mortality in the general population (independent from HF), pre-existing HF prevalence, incidence, and respective survival [12]. Declines in mortality in general could reduce the HF IBM, reducing the pre-existing prevalence and incidence can reduce the number of patients with HF, and increasing the survival can reduce the deaths. For example, the incidence of HF has decreased despite the increases in the absolute number of patients with HF due to the population aging [13]. Divergent trends in incidence of HF subtypes have been observed with decreases in HF with reduced ejection fraction and increases in HF with preserved ejection fraction [14,15]. Further, the US hospitalization for HF decreased up until 2012, and increased during 2013‐2017, which may exert effects on patient survival after HF diagnosis [16]. To facilitate the understanding of the dynamics in HF, it is important to partition the prevalence and mortality into their underlying determinants and understand the respective directions and magnitude from the overall trends.

In this study, we used 5% of US Medicare claims data to investigate the relative contributions of epidemiologic determinants to the overall trends of HF prevalence and IBM using an innovative partitioning approach. We believe the findings of this study will provide evidence regarding the changes in the direction and magnitude of each determinant’s contribution that enables us to identify modifiable factors to be intervened for HF.

## Methods

### Data Source

This study was based on administrative claims data (1992‐2017) drawn from a nationwide representative 5% sample of the US Medicare beneficiaries. Medicare is a federal health insurance program for adults aged 65+ years as well as people with specific disabilities and end-stage renal disease [17]. The data cover beneficiaries’ encounters with the health care system and the receipt of treatment and intervention (eg, procedures, medications, or services), and can be used to examine morbidity, mortality, burden of disease, effectiveness of therapies, cost of care, effect of policy change, etc. The 5% random sample is generated annually by the Centers for Medicare & Medicaid Services from the condensed enrollment database of the entire Medicare population based on specific numerical sequences (5, 20, 45, 70, or 95) appearing in the eighth and ninth positions of the enrollee’s health insurance claim number. Researchers could submit their proposals to Centers for Medicare & Medicaid Services via the Research Data Assistance Center to apply for claims data for epidemiologic and health outcome research. The Research Data Assistance Center will review the scientific merit and technical feasibility of the submitted request. If approved, researchers will obtain access to the data after entering a data use agreement. In this study, individuals whose Medicare coverage was less than 20% of their months since they became 65 years old were excluded. HF diagnosis was identified using the *ICD-9* (*International Classification of Diseases, Ninth Revision*) code 428 and *ICD-10* (*International Classification of Diseases, Tenth Revision*) code I50. This was a secondary data analysis.

### Measures

Age-adjusted prevalence of HF was estimated based on the incident cases identified through the Medicare records. Age-adjusted IBM was defined as the all-cause mortality that occurred in patients with HF. HF incidence was defined by the earliest record of HF with a respective *ICD-9*/*ICD-10* code in the Medicare records that was confirmed by the second record with HF diagnosis within 0.3 years [18,19].

According to the partitioning approach developed by our team [11,12], the constituent components of age-adjusted prevalence and IBM included a pre-existing prevalence at age 65 years (the observation age of our study begins), its pre-existing prevalence at the calendar year 1992 (the observation year of our study begins), disease incidence, relative survival, and mortality in the general population (used for IBM partitioning only). Pre-existing prevalence at age 65 years was defined based on the number of prevalent cases in patients diagnosed with HF before age 65 years and those who survived to the age 65 years. These patients will contribute to the prevalence over this study’s period but must be treated differently as the exact age of onset cannot be identified. Similar logic requires separate treatment of prevalent individuals of any age entering the data at its calendar year boundary (1992). HF incidence and survival were defined based on new HF cases diagnosed after age 65 years and the year 1992 and their respective survival thereafter. Mortality in the general population represented the all-cause mortality independent of HF.

### Statistical Analysis

**Figure 1.**
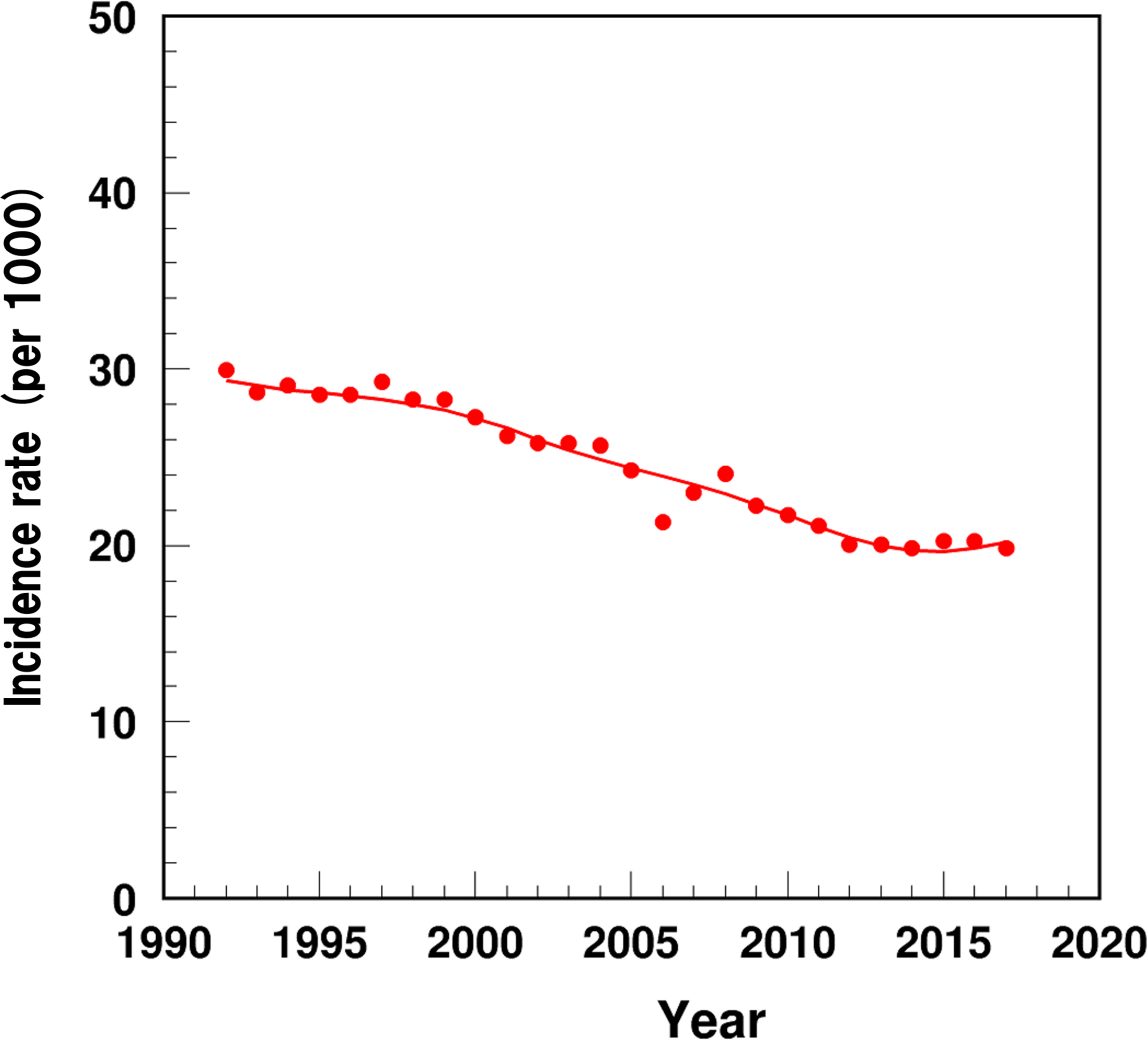
Temporal trend of age-standardized heart failure incidence among US adults aged 66‐99 years, 1991‐2017: empiric estimates (dots) and model-based estimates (solid line).

The detailed algorithms for HF outcomes are described in the Multimedia Appendix 1. The partitioning approach used in this study was described in our team’s publications [11,12]. Briefly, the partitioning analysis was used to decompose the dynamics in HF prevalence and IBM into their constituent determinants (eg, pre-existing prevalence, incidence, or survival). We compared the magnitude and direction of the impacts of each determinant on the dynamics in the time trends of HF prevalence and IBM. The empirically estimated rates of age-adjusted HF incidence and 1-, 3-, and 5-year survival were compared with the model-estimated rates, with a high agreement between the two indicating good model fit (Figures1  2).

**Figure 2.**
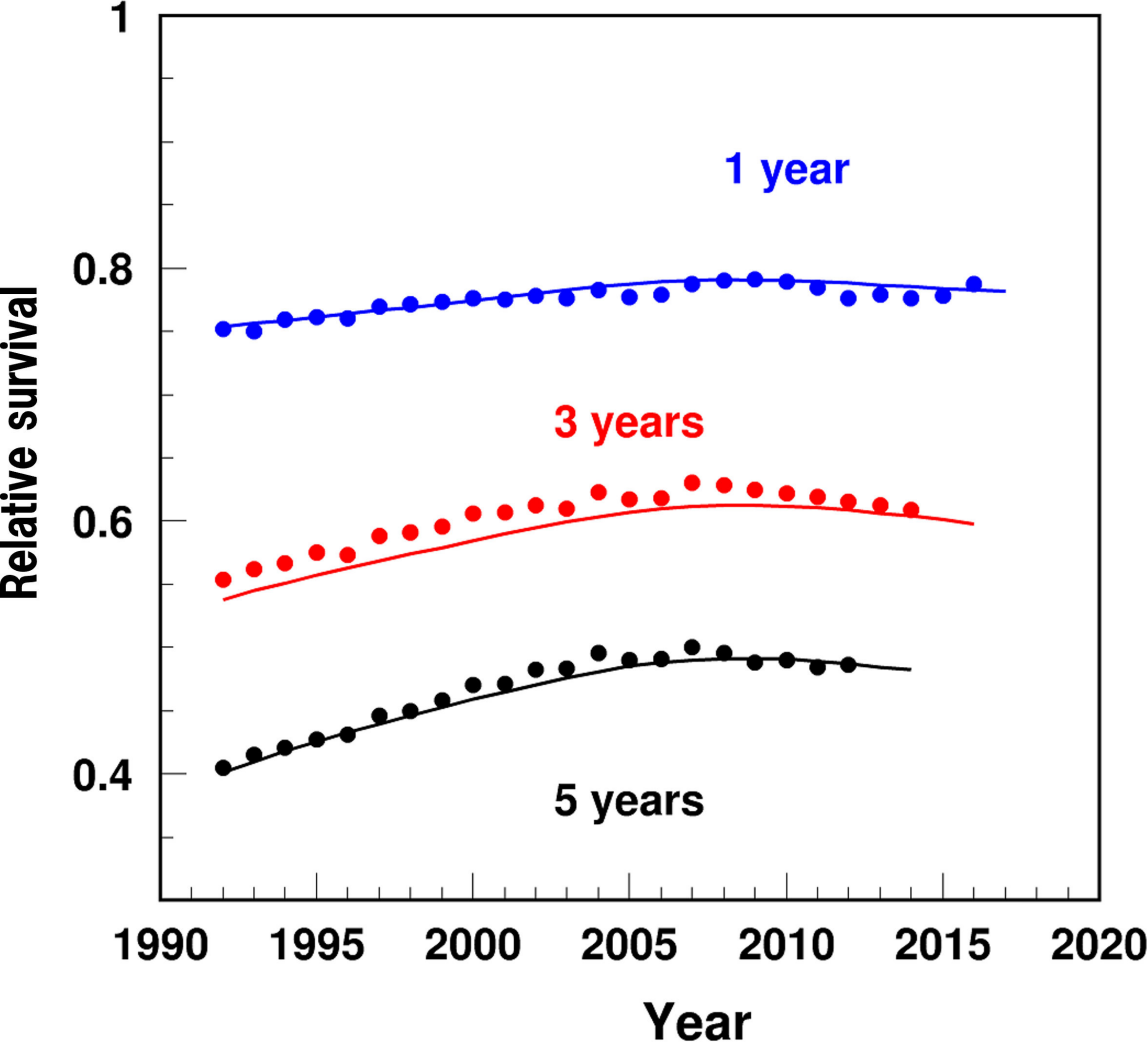
Temporal trend of age-standardized 1-, 3-, and 5-year survival rates after heart failure diagnosis among US adults aged 66‐99 years, 1991‐2017: empiric estimates (dots) and model-based estimates (solid line).

Theoretically, there are three components that contribute to HF prevalence (P(y)), including patients with HF being diagnosed (1) before patients’ age 65 years ($P_{0}(y)$), (2) before the year 1992 ($P_{00}(y)$), and (3) after age 65 years and year 1992 ($P_{is}(y)$; Equation 1).


(1) $$P\left(y\right)=P_{0}\left(y\right)+P_{00}\left(y\right)+P_{is}\left(y\right)$$

The change in prevalence is then modeled using the first derivative of the above functions (Equation 2):


(2) $$P_{y}^{`}\left(y\right)/P_{y}(y)=T_{0}\left(y\right)+T_{00}\left(y\right)+T_{inc}\left(y\right)+T_{s}(y)$$

where $P_{y}^{\prime }(y)/P_{y}(y)$ denotes the first derivative of the age-adjusted prevalence, $T_{0}(y)$ denotes the contribution from the prevalence of patients diagnosed before age 65 years and their relative survival, $T_{00}(y)$ denotes the contribution from the prevalence of patients diagnosed before the year 1992 and their relative survival, $T_{inc}(y)$ denotes the contribution from the incidence of patients diagnosed after age 65 years and year 1992, and $T_{s}(y)$ denotes the contribution from survival after HF diagnosis for patients diagnosed after age 65 years and year 1992.

Likewise, IBM (M(y)) decomposition consists of four components (Equation 3), including (1) mortality in the general population ($M_{mu}(y)$), (2) mortality for patients diagnosed before age 65 years ($M_{0}(y)$), (3) mortality for patients diagnosed before the year 1992 ($M_{00}(y)$), and (4) mortality for patients diagnosed after age 65 years and year 1992 ($M_{is}(y)$).


(3) $$M\left(y\right)=M_{mu}\left(y\right)+M_{0}\left(y\right)+M_{00}\left(y\right)+M_{is}(y)$$

The first derivative of the IBM function is then used to model change in IBM as shown in Equation 4:


(4) $$M_{y}^{\prime }\left(y\right)/M_{y}\left(y\right)={\hat{T}}_{mu}\left(y\right)+{\hat{T}}_{P}\left(y\right)+{\hat{T}}_{0}\left(y\right)+{\hat{T}}_{00}\left(y\right)+{\hat{T}}_{inc}\left(y\right)+{\hat{T}}_{S}\left(y\right)$$

where $M_{y}^{\prime }(y)/M_{y}(y)$ denotes the derivative of mortality, ${\hat{T}}_{mu}(y)$ denotes contribution from the mortality in the general population, ${\hat{T}}_{P}(y)$ denotes contribution from the disease prevalence, ${\hat{T}}_{0}(y)$ denotes contribution from the mortality of patients diagnosed before being aged 65 years, ${\hat{T}}_{00}(y)$ denotes contribution from the mortality of patients diagnosed before the year 1992, ${\hat{T}}_{inc}(y)$ denotes contribution from the incidence of patients, and ${\hat{T}}_{S}(y)$ denotes contribution from the survival.

To simplify the equations, ${\hat{T}}_{P}(y)$ in Equation 4 can be split into its components as per Equation 2 and joined with the incidence and survival components already present, resulting in a more parsimonious equation (Equation 5) [14].


(5) $$M_{y}^{\prime }\left(y\right)/M_{y}\left(y\right)={\hat{T}}_{mu}\left(y\right)+{\tilde{T}}_{0}\left(y\right)+{\tilde{T}}_{00}\left(y\right)+{\tilde{T}}_{inc}\left(y\right)+{\tilde{T}}_{S}\left(y\right)$$

**Figure 3.**
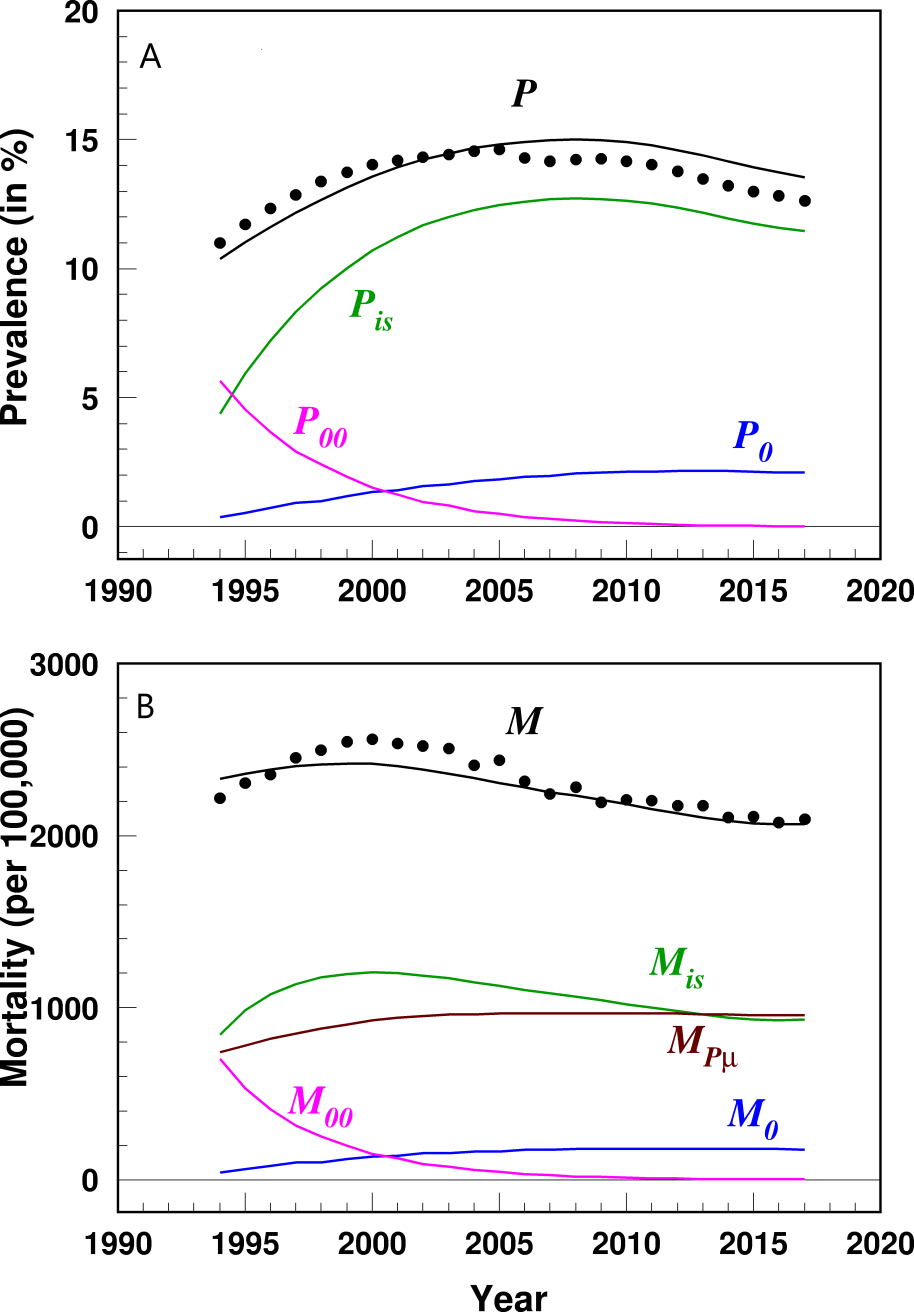
Temporal trends of heart failure prevalence (A) and incidence-based mortality (B) and contribution from their partitioned determinants among US adults aged 66‐99 years, 1991‐2017. Data were derived from the 5% sample of Medicare beneficiaries. The variables are defined as follows: *P*: prevalence; $P_{0}$: contribution from prevalence of patients diagnosed before being aged 65 years; $P_{00}$: contribution from prevalence of patients diagnosed before year 1992; $P_{is}$: contribution from prevalence of patients diagnosed after being aged 65 years and year 1992; M: incidence-based mortality; $M_{0}$: contribution from mortality of patients diagnosed before being aged 65 years; $M_{00}$: contribution from mortality of patients diagnosed before year 1992; $M_{is}$: contribution from mortality of patients diagnosed after being aged 65 years and year 1992; $M_{Pu}$: contribution from mortality in the general population.

**Figure 4.**
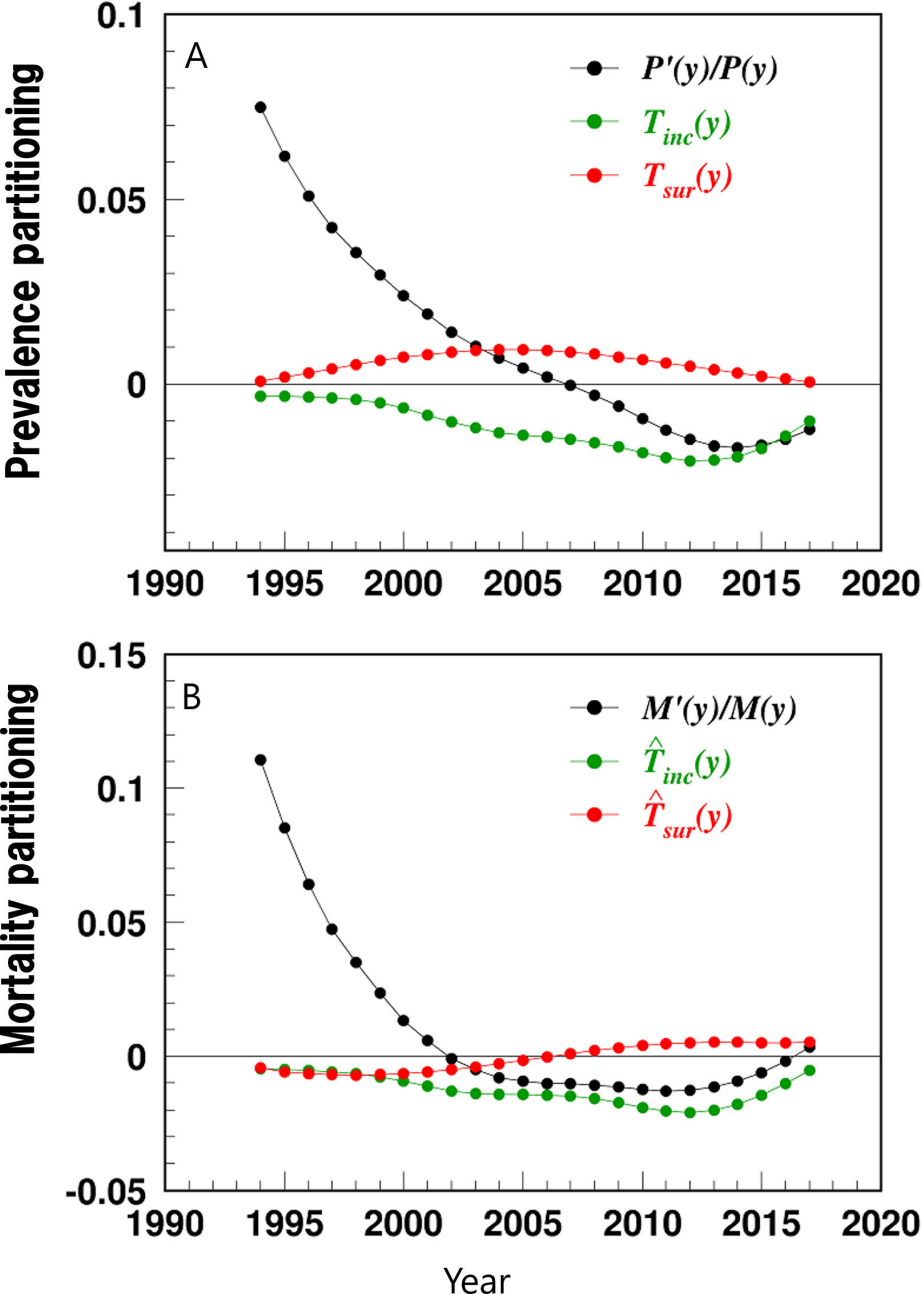
Temporal trends of derivatives of heart failure prevalence (A) and incidence-based mortality (B) and their partitioned determinants among US adults aged 66‐99 years, 1991‐2017. Data were derived from the 5% sample of Medicare beneficiaries. The variables are defined as follows: $P^{\prime }(y)/P(y)$: derivative of prevalence; $T_{inc}(y)$: prevalence contribution from incidence; $T_{sur}(y)$: prevalence contribution from relative survival; $M^{\prime }(y)/M(y)$: derivative of mortality; ${\hat{T}}_{inc}(y)$: mortality contribution from incidence; ${\hat{T}}_{sur}(y)$: mortality contribution from the relative survival.

The temporal trends of the results of the partitioning analysis for HF prevalence and IBM were then plotted, including (1) HF prevalence and contribution from its determinants (Equation 1, Figure 3A), (2) derivative of prevalence and contribution from its partitioned determinants (Equation 2, Figure 4A), (3) IBM and contribution from its determinants (Equation 3, Figure 3B), and (4) derivative of IBM and contribution from its determinants (Equation 5, Figure 4B). If the derivative is >0, then the respective rate of prevalence or mortality is increasing, and vice versa. The magnitude of the derivative represents the speed of change with a higher derivative indicating quicker increase or decline. The partitioning results of contributions from incidence and survival were shown in the main results, while the partitioning results for other determinants were included in Figure S1 in Multimedia Appendix 1. The proportion of the relative contribution from each partitioned determinant was also estimated as the value of the specific determinant divided by the derivative of the prevalence or mortality, with a positive or negative percentage indicating whether the contribution of that determinant has the same or opposite direction as the derivative of prevalence or mortality (Table 1). All analyses were performed using SAS software (version 9.4; SAS Institute, Inc).

**Table 1.** Contribution from partitioned determinants of heart failure prevalence and incidence-based mortality (%).

Component	1994	1996	1998	2000	2002	2004	2006	2008	2010	2012	2014	2016	2017
Prevalence													
$T_{0}(y)$^a^	2.8	7	10.8	19.2	30	52.6	149.2	−69.3	−12.1	0.47	7	16	24
$T_{00}(y)$^b^	100.3	93.7	85.7	77.2	81.4	101.6	216.1	−84	−15.7	−6.4	−3	−1.8	−1.3
$T_{inc}(y)$^c^	−4.3	−6.7	−11.6	−26.9	−72.6	−186.8	−722.6	521.5	199.2	138.5	113.8	95.1	82.2
$T_{sur}(y)$^d^	1.1	6	15	30.5	61.2	132.6	457.3	−268.2	−71.4	−32.6	−17.7	−9.2	−4.8
Mortality													
${\hat{T}}_{mu}(y)$^e^	−0.35	−1.44	−5.33	−24.1	513.4	63.2	45.2	28.1	6.5	−8.3	−25.7	−181.4	97.1
${\hat{T}}_{0}(y)$^f^	0.65	2.1	4.3	14.2	−178.2	−14.8	−8	−4.7	−2.4	0.5	3.2	35.5	−20.8
${\hat{T}}_{00}(y)$^g^	107.9	117.7	139.7	226.8	−2222.1	−161.3	−82	−48.3	−25.8	−17.6	−14	−41.6	13.6
${\hat{T}}_{inc}(y)$^h^	−4.2	−8.2	−18.7	−68.8	1427.7	177.4	142.1	146	155.3	166.1	193.5	584.7	−151.5
${\hat{T}}_{sur}(y)$^i^	−3.9	−10.2	−19.9	−48.1	559.1	35.6	2.7	−21.1	−33.5	−40.7	−56.9	−297.2	161.6

a Prevalence contribution from patients diagnosed before age 65.

b Prevalence contribution from patients diagnosed before year 1992.

c Prevalence contribution from incidence.

d Prevalence contribution from relative survival.

e Mortality contribution from the mortality in the general population.

f Mortality contribution from patients diagnosed before age 65.

g Mortality contribution from patients diagnosed before the year 1992.

h Mortality contribution from the incidence of patients.

i Mortality contribution from the survival.

### Ethical Considerations

Patients or the public were not involved in the design, conduct, reporting, or dissemination plans of our research. All data analyses were designed and performed in accordance with the ethical standards of the responsible committee on human studies and with the Declaration of Helsinki (of 1975, revised in 2013), and all were approved by Duke University Health System Institutional Review Board for Clinical Investigations; written informed consent for participation was not required for this study in accordance with the national legislation and the institutional requirements (IRB FWA00009025).

## Results

### Empiric and Modeled Estimates of HF Incidence and Survival

The empiric HF incidence rates (1/1000 person-years) declined from 29.9 in 1992 (the highest) to 19.9 in 2017 (the lowest; Figure 1). The model estimates of HF incidence fitted the empiric estimates with high precision.

Further, the 1-year survival rate increased from 76% in 1994 to 79.2% in 2009, followed by a decline to 78.8 in 2016 (Figure 2). The survival rates varied from 56.7% in 1994 to 60.9% in 2014 for 3-year survival, and from 42.1% in 1994 to 48.6% in 2012 for 5-year survival. The model estimates of survival fitted the empiric estimates with high precision.

### Partitioning of HF Prevalence

The age-adjusted HF prevalence (P(y), 1/100 person-years) increased from 11 in 1994 to 14.6 in 2005, followed by a decline to 12.6 in 2017 (Figure 3A). During the same study period, the contribution from patients diagnosed with HF before being aged 65 years ($P_{0}(y)$) increased from 0.4 to 2.1, while the contribution from patients diagnosed before 1992 ($P_{00}(y)$) declined from 5.6 to 0.01 and the contribution from patients diagnosed after the age of 65 years and year 1992 ($P_{is}(y)$) increased from 4.4 to 11.5.

Further, 3 phases of dynamics in HF prevalence were identified (Figure 4A and Table 1). The first phase was a decelerated increasing prevalence (1994‐2006). During this period, the declining incidence drove the decelerated increasing prevalence (eg, −72.6% in 2002), overpowering the increasing survival (eg, 61.2% in 2002).

The second phase was an accelerated declining prevalence (2007‐2014). During this period, the declining incidence drove the decreases in prevalence (eg, 138.5% in 2012), overpowering the increase in prevalence contributed from survival (eg, −32.6% in 2012).

The third phase was a decelerated declining prevalence (2015‐2017). In this period, the declining incidence persisted, driving the declines in prevalence (eg, 95.1% in 2016), overpowering the prevalence increase contributed from the survival (eg, −9.2%). Due to the leveling-off of the declining trend in incidence, the decline in prevalence decelerated.

The contributions from other epidemiologic determinants were relatively small (Figure S1 in Multimedia Appendix 1 and Table 1). For example, contributions from patients diagnosed before age 65 years and before year 1992 in 2013 were 3.4% and −3.9%, respectively.

### Partitioning of HF Mortality

The age-adjusted HF IBM (1/100,000) increased from 2220.8 in 1994 to 2563.7 in 2000, then declined to 2075.9 in 2016 followed by an increase to 2094.7 in 2017 (Figure 3B). HF IBM consisted of four components: (1) contribution from patients diagnosed before age 65 years ($M_{0}(y)$) varied from 42.7 in 1994 to 176.6 in 2017, (2) contribution from patients diagnosed before year 1992 ($M_{00}(y)$) varied from 704.3 to 1.2, (3) contribution from patients diagnosed after age 65 years and year 1992 ($M_{is}(y)$) increased from 845.2 to 934, and (4) contribution from mortality in the general population ($M_{mu}(y)$) increased from 740.9 to 957.4.

The dynamics of HF IBM can be divided into 3 phases (Figure 4B and Table 1). The first phase was a decelerated increasing mortality (1994‐2001). During this phase, the declining incidence (eg, −18.7% in 1998) and increasing survival (eg, −19.9% in 1998) decelerated the mortality increases.

The second phase was an accelerated declining mortality (2002‐2012). In this phase, the declining incidence in 2002‐2012 (eg, 155.3% in 2010) and the increasing survival in 2002‐2006 (eg, 35.6% in 2004) contributed to the mortality declines, but the declining survival in 2007‐2012 contributed to the increases in mortality (eg, −33.5% in 2010). During 2007‐2012, the effect from incidence overpowered the effect from survival.

The third phase was a decelerated declining mortality (2013‐2017). During this phase, the declining incidence drove the declines in mortality (eg, 193.5% in 2014), overpowering the declining survival (eg, −56.9% in 2014) that drove the increases in mortality. Due to the leveling-off in HF incidence decline, the declining mortality decelerated.

The contributions from other partitioned determinants are relatively small (Figure S1 in Multimedia Appendix 1 and Table 1). For example, in 2012, the relative contributions from patients diagnosed before age 65 years and before year 1992 and from mortality in the general population were 0.5%, −17.6%, and −8.3%, respectively.

## Discussion

### Principal Findings

This study used partitioning analysis to investigate time trends of HF prevalence and mortality among US older adults. Findings of this study revealed decade-long declines in HF prevalence and mortality that were mainly caused by declining incidence, and a most recent increase in mortality that was driven by the alarming declining survival. Compared with previous studies using death registries data that only showed the temporal trend of mortality [20,21], findings of this study provide valuable information for researchers, health professionals, and policy decision makers to understand the causes of the dynamics of HF prevalence and mortality, and provide evidence regarding the modifiable determinants in curbing the forecasted increasing trend of HF prevalence and mortality.

HF prevalence increased during 1992‐2005 and slightly declined thereafter, which was consistent with the previous studies [22,23], while HF mortality increased in 1994‐2000 and declined to 2016, and reversed in 2017. The decade-long declines in HF prevalence and mortality were mainly attributable to the beneficial effects from the decreasing incidence and increasing survival, suggesting the effectiveness of the previous public health prevention programs targeting HF risk factors and advancements in clinical treatment and management in patients with HF [23,24]. However, the leveling-off in the decreasing HF incidence and declines in survival among patients with HF in recent years may suggest harmful effects on HF prevalence and mortality. Studies from Europe [25] and Asia [26] also reported declining incidence and increasing prevalence of HF.

During this study’s period, the declining HF incidence contributed to the declines in HF prevalence and mortality. The declining incidence, consistent with previous studies from North America [22,23], Europe [25], and Asia [26], may have resulted from the successful decade-long prevention of modifiable risk factors, including tobacco and alcohol use, and improvements in awareness, treatment, and management of cardiovascular diseases and comorbidities [8,23]. However, due to the leveling-off in HF incidence in recent years, which may be attributable to the increasing prevalence of obesity and unhealthy lifestyles (eg, low physical activity) [3], the declines in HF prevalence and mortality decelerated. That suggests the need of public health efforts in prevention of these relatively new risk factors. It is also likely that the increasing application of noninvasive testing techniques (eg, echocardiography [27,28]) may contribute to the recent increases in HF incidence, which is beneficial for the early diagnosis and early treatment. We observed an increase in HF incidence in 2006‐2008, which may be associated with the declines in echocardiography charges and increases in number of procedures [28].

Study results also showed that in early years, the survival after HF diagnosis was increasing, which may have benefited from the improvements in awareness, treatment, and management of HF and its related comorbidities [23,24], driving the increases in prevalence and decline in mortality. However, the survival started to decline in recent years, and the contribution from the survival on prevalence declined as well, although it still showed a beneficial effect driving the prevalence increase. The time lag between the declining survival and the declining but still beneficial effect of survival on prevalence may be associated with the accumulated survivors from previous years (ie, with the passage of these survivors over time, the contribution on prevalence was declining). For mortality, the recent declining survival contributed to the harmful effects on HF mortality, and this effect overpowered the beneficial effect from the declining incidence in 2017, resulting in a 1-year increase in mortality. The harmful survival effect on mortality may be also related with the accumulated survivors from previous years (ie, these survivors may have higher mortality that was due to the aging process with comorbidities) [29].

The recent declines in survival revealed in this study may be associated with the Hospital Readmissions Reduction Program (HRRP; discussed in 2007‐2009, announced in 2010, and implemented in 2012) [30,31]. The initial purpose of the HRRP was to improve the hospitals’ care quality in reducing the readmission rates by putting penalties on hospitals with higher-than-expected readmission rates [32]. The HRRP initially targeted HF, acute myocardial infarction, and pneumonia in 2012 [31]. However, the HRRP may have led the hospitals to take inappropriate actions to avoid or reduce the penalties, such as delaying patients’ readmission beyond day 30, increasing observation stays, shifting inpatient care to emergency care [33], or increasing the coding disease severity [34], which may adversely affect the health outcomes in patients with HF [33]. Several studies indicated that the HRRP may be associated with the HF mortality increase [35,36].

In addition to incidence and survival, other epidemiologic determinants also contributed to the dynamics in HF prevalence and mortality. The contribution from patients diagnosed before year 1992 on prevalence and mortality declined substantially along with the years, which was due to the passage of these patients [29,37]. The contribution from patients diagnosed before age 65 years contributed to the declines of the prevalence and mortality in recent years, suggesting the beneficial effects from the early diagnosis and early treatment. The mortality in the general population contributed to the increase in HF IBM, suggesting the increases in mortality from other diseases that are independent from HF. Data from CDC WONDER (Wide-Ranging Online Data for Epidemiologic Research) showed the increases in non–HF-related diseases among older adults, including suicide, substance abuse-related deaths, mental health disorders, unintentional injuries, Alzheimer disease, and Parkinson disease [38].

Our study results can also be used to forecast the future trends of HF outcomes. If the current trends of incidence and survival persist, we would expect to see increases in HF prevalence and mortality. Thus, it is urgent to take actions for prevention strategies in decreasing the incidence by intervening the HF risk factors and improving survival by developing better HF treatment and management.

This study has strengths. First, the data used in this study were from Medicare, which is a representative sample of adults aged older than 65 years in the United States; thus, findings of this study could be generalized to the broader US older adult population. Second, Medicare is a large dataset (nearly 64 million as of 2021) containing longitudinal information on health care service, which can help accurately define the onset of HF. Third, withdrawal from Medicare is extremely rare, as once individuals are enrolled in Medicare, they are typically followed until death. Fourth, Medicare data reflect near-complete capture of health care services across all settings of care; thus, they can be used to estimate the prevalence, incidence, survival, and mortality indicators. Last but not the least, the partitioning analysis approach has been well validated in multiple previous studies published in prestigious journals. Meanwhile, this study has limitations. First, all calculations in this study are based on empiric parametric models or widely accepted modeling approaches that involve the models for age-dependent incidence and age- and time-dependent survival after diagnosis, but statistical uncertainties still exist [29]. Second, information on specific subtypes of HF is limited in Medicare data; thus, caution is needed when generalizing the findings of this study to individual HF subtypes.

### Conclusion

Findings of this study showed recent decade-long declines in HF prevalence and mortality, largely reflecting the declines in incidence, and a most recent increase in HF mortality that was primarily caused by the alarming declines in survival. HF prevalence and mortality are forecasted to increase if the current trends in HF incidence and survival persist. Prevention strategies should primarily focus on the improvement of treatment and management of HF after diagnosis and continue the prevention of HF risk factors (eg, obesity or low physical activity).

## Supplementary material

10.2196/51989 Multimedia Appendix 1 Figures, notes, and equations on Medicare.

## Abbreviations

CDC: US Centers for Disease Control and Prevention
HF: heart failure
HRRP: Hospital Readmissions Reduction Program
IBM: incidence-based mortality
*ICD-10*: *International Classification of Diseases, Tenth Revision*
*ICD-9*: *International Classification of Diseases, Ninth Revision*
WONDER: Wide-Ranging Online Data for Epidemiologic Research
