# Supplementary material for: Decomposition of Heart Failure Prevalence and Mortality Among Older Adults in the United States: Medicare-Based Partitioning Analysis
Source: JMIR Public Health Surveill. 2024 Nov 20;10:e51989. doi: 10.2196/51989 (PMC11611790; doi:10.2196/51989)
Supplement: Multimedia Appendix 1 [file publichealth-v10-e51989-s001.docx]

**Results**

**Figure S1.** Temporal trends of derivatives of heart failure prevalence and incidence-based mortality and their partitioned determinants among US adults aged 66-99 years old, 1991-2017.

Note: Data were derived from the 5% sample of Medicare Beneficiaries. ${P^{'}(y)}/{P(y)}$ = Derivative of prevalence, : $T_{0}(y)$ = Prevalence contribution from patients diagnosed before age 65, $T_{00}(y)$ = Prevalence contribution from patients diagnosed before year 1992, $T_{inc}(y)$ = Prevalence contribution from incidence, $T_{sur}(y)$ = Prevalence contribution from relative survival, ${M^{'}\left( y \right)}/{M\left( y \right)}$ = Derivative of mortality, $\hat{T}_{0}(y)$ = Mortality contribution from patients diagnosed before age 65, $\hat{T}_{00}(y)$ = Mortality contribution from patients diagnosed before year 1992, $\hat{T}_{inc}(y)$ = Mortality contribution from incidence, $\hat{T}_{sur}(y)$ = Mortality contribution from the relative survival, $\hat{T}_{mu}(y)$ = Contribution from mortality in the general population.

**Supplementary Methods:**

Medicare data and ascertainment algorithm ^[1]^ results in the following variables for each eligible individual: $a_{ini}^{(i)}$ –– initial age of follow-up, $a_{fin}^{(i)}$ –– final age of follow-up, $\delta_{d}^{(i)}$ –– death/censoring indicator ($\delta_{d}^{(i)}=1$ for death and $\delta_{d}^{(i)}=0$ for censoring) at age $a_{fin}^{(i)}$; $a_{HF}^{(i)}$–– age of HF diagnosis (or missing). The superscript $(i)$ denotes individual characteristics. For each individual, dates of birth $y_{b}^{(i)}$ are available allowing us to calculate exact age at any point of the individuals’ follow-up. An individual is considered prevalent at the time of Medicare enrollment if there exists a record with the HF code during 1 year after enrollment; $\delta_{p1}^{(i)}$ is an indicator of prevalence at the time of Medicare enrollment where $\delta_{p1}^{(i)}=1$ for prevalent and $\delta_{p1}^{(i)}=0$ for non-prevalent individuals; $\delta_{p0}^{(i)}=1-\delta_{p1}^{(i)}$ is an indicator of being non-prevalent at the time of Medicare enrollment.

We calculate prevalence, incidence rates, incidence-based mortality, and all-cause mortality in age- and time- bins (representing rectangles in the age and time plain where the Medicare data are available, Figure S2). Specifically, the measures are calculated in 29 age groups including 25 one-year groups between ages 65 and 90, and three other groups: 90-91, 92-94, 95-99, 100-110. Person-years after age 110 are considered invalid and ignored. Similarly we consider one-year calendar time groups in the period between year $y_{ini}$ and $y_{fin}$. Thus, all age-specific measures are two-dimensional and calculated in age and time groups.

**Figure S2.** Conceptual visualization of the age- and time- bins in Medicare data.

An individual trajectory in this plain (also shown in Figure S2) contributes to different rectangle bins during each individual follow-up period. Age-specific prevalence, incidence, incidence-based mortality, and all-cause mortality in a certain rectangle bin are calculated through the ratio $N_{c}^{meas}/N_{py}$ , where $N_{c}^{meas}$ is the number of effective cases that are measure specific (i.e., $meas=inc$ for incidence, $meas=prev$for prevalence, $meas=mort$for incidence-based mortality, and $meas=allc$for all-cause mortality). The number of person-years $N_{py}$ is identical for all measures and calculated as:

$$N_{py}=\sum_{i} \left(y_{2}^{(i)}-y_{1}^{(i)}\right)$$

where $y_{1}^{(i)}$ and $y_{2}^{(i)}$are the initial and final individual times for an age group with bounds defined by ages $a_{1}$ and $a_{2}$ in a time group with bounds $y$ and $y+1$ :

$$\begin{matrix} y_{1}^{(i)}=\max\left(y-y_{b}^{(i)},a_{ini}^{(i)},a_{1}\right) \\ y_{2}^{(i)}=\min\left(y+1-y_{b}^{(i)},a_{fin}^{(i)},a_{2}\right) \end{matrix}$$

The numbers of effective cases are measure-specific:

$$\begin{matrix} N_{c}^{prev}=\sum_{i} \left(\delta_{p1}^{(i)}\left(y_{2}^{(i)}-y_{1}^{(i)}\right)+\delta_{p0}^{(i)}I\left(0\leq a_{HF}^{(i)}<y_{2}^{(i)}\right)\max(0,y_{2}^{(i)}-\max(a_{HF}^{(i)},y_{1}^{(i)})\right) \\ N_{c}^{inc}=\sum_{i} \delta_{p0}^{(i)}I\left(y_{1}^{(i)}<a_{HF}^{(i)}\leq y_{2}^{(i)}\right) \\ N_{c}^{mort}=\sum_{i} \delta_{d}^{(i)}I\left(y_{1}^{(i)}<a_{fin}^{(i)}\leq y_{2}^{(i)}\right)I\left(0\leq a_{HF}^{(i)}<a_{fin}^{(i)}\right) \\ N_{c}^{allc}=\sum_{i} \delta_{d}^{(i)}I\left(y_{1}^{(i)}<a_{fin}^{(i)}\leq y_{2}^{(i)}\right) \end{matrix}$$

These formulas are illustrated in Figure S3.

**Figure S3.** Conceptual visualization of the formulas in the study.

Reference

^[1]^ Akushevich I, Kravchenko J, Ukraintseva S, Arbeev K, Yashin AI. Age Patterns of Incidence of Geriatric Disease in the US Elderly Population: Medicare‐Based Analysis. *Journal of the American Geriatrics Society.* 2012;60(2):323-327.
